# A bibliometric analysis of investigations of polybrominated diphenyl ethers (PBDEs) in biological and environmental matrices from 1992 – 2018

**DOI:** 10.1016/j.heliyon.2018.e00964

**Published:** 2018-11-26

**Authors:** Chijioke Olisah, Omobola O. Okoh, Anthony I. Okoh

**Affiliations:** a Department of Pure and Applied Chemistry, University of Fort Hare, Alice 5700, South Africa; b Applied and Environmental Microbiology Research Group (AEMREG), Alice 5700, South Africa; c SAMRC, Microbial Water Quality Monitoring Centre, University of Fort Hare, Alice 5700, South Africa

**Keywords:** Analytical chemistry, Organic chemistry, Environmental science

## Abstract

The aim of this bibliometric analysis is to review the status and research evolution on the analysis of polybrominated diphenyl ethers (PBDEs) on biological and environmental matrices from January 1992 to February 2018 in the Web of Science focusing on original articles and reviews. One thousand four hundred and eighty two articles were found in the databases of the Web of Science on the analysis of PBDEs. Quantitative and qualitative parameters (countries, number of articles, frequency, average article citations and total average citations) were used to analyse each article and ranking of countries based on productivity, authors and article citation. Complementary analysis based on keywords was also done. The last decade experienced an increase in the analysis of this pollutant with the year 2012 recording the highest number of published articles (n = 137). High rate of collaboration with a very rich research network exists amongst institutions in Asian, European and America countries. China and USA are ranked 1^st^ and 2^nd^ on countries based on productivity, publishing 30% and 21.7% of the total articles respectively. South Africa was the only African country found in the category of countries based on productivity occupying the 17^th^ position. The spectacular growth of research by researchers domiciled in China suggests the dominance of China in scientific research. This study suggests high research interest on this class of pollutant in developed countries. Additionally, lack of funds and sophisticated analytical tools may be responsible for lack of PBDEs-related studies in developing countries especially in Africa.

## 1 Introduction

Over the years, the increase of research activities in the various institutions in the world has led to an increase in investment from various funding agencies in many countries most especially the People's Republic of China and the USA (Cañas-Guerrero et al., 2013). The steady increase of scientific studies has given importance to bibliometric analysis (Quevedo-Blasco and Lopez-Lopez, 2010). This tool uses a statistical method to analyse books, articles, subject literature, publications, trends of research and others kinds of bibliographies and reports (Lawani, 1981). This kind of analysis assists researchers in each discipline to streamline their choice of articles or journal to a particular subject matter. It also provides help to the scientist who has various journals to read and refer to, giving them an idea of where to publish their articles (Wang et al., 2010).

The problem of choice can easily be sorted out using bibliometric databases which can be assessed using a software installed on a computer system. With this, it is easier to gain access to these databases which are made up of data compilation designed on an electronic support (Pradham, 2016). The Web of Science (WoS) (ISI, Thomson Reuters), which comprised of seven databases, has been preferred for this study, due to its broad range of information dissemination in various disciplines (de Granda-Orive et al., 2011; Chirici, 2012). More than 2000 publications on bibliometric analysis exist in the database of the WoS analysing different research fields (Molatudi and Pouris, 2006; Narotsky et al., 2012; Cañas-Guerrero et al., 2013). In this study, the bibliometric analysis was used to analyse the category “Polybrominated Diphenyl Ethers (PBDEs)”. This class of contaminants are found in various polymeric and electrical materials as fire preventing substances (Law et al., 2006). Due to their ubiquitous nature, they accumulate in the environment with a great ability to transport long-range. Also, their high affinity for lipids has made it possible for them to be found in significant concentration in biota (De Wit, 2002; Covaci et al., 2006). The Penta and Octa-formulate of Polybrominated diphenyl ethers (PBDEs) has been banned worldwide (Directive EEC, 2003) while the European Union (EU) banned the use of Deca-BDE formulates in electrical appliances in 2008 (European Court of Justice, 2008).

Various studies have been carried out on the analysis of this pollutant in different environmental matrices in different regions of the world and most of these studies have been published in peer review journals. However, to the best of our knowledge, there is paucity of data on bibliometric analysis of this pollutant class and organic contaminants in general. Also, because these pollutants have been widely studied, there is a need for its bibliometric compilation. The principal objectives of this study are to carry out a global analysis of the research activities on PBDEs from January 1992 to February 2018 from research articles available for downloads in the databases of WoS, studying the research progression in the previous year's using bibliometric analyses techniques. In order to achieve this, various quantitative parameters were used to clearly analyse these data. These include number of publications, most productive researchers, international collaboration, most frequent cited manuscripts, most productive countries and affiliations, total citations per country, most relevant sources, most relevant keywords, and most cited authors. Furthermore, in this article, special attention was paid to scientific journals which published articles on the analysis of these pollutants and their relationship with the most productive countries.

## 2 Materials and methods

Published studies on polybrominated diphenyl ethers (PBDEs) were retrieved from the WoS database on February 5, 2018. The WoS hosted reliable and comprehensive high-impact scientific studies (Zyoud et al., 2017). We used the key term “polybrominated diphenyl ethers” as identifier of studies published between January 1 1992 and February 1, 2018, using title-specific search to improve recovery and specificity. A title-specific search has merit of significant recovery, minimal loss of sensitivity and specificity over a topic search (Aleixandre et al., 2015; Sweileh et al., 2016; Zyoud et al., 2017). The search results were further streamlined to original articles and reviews excluding all other document types such as meeting abstract, letters and proceeding paper. These two parameters were selected because they are generally considered as an original contribution to science (Moed, 2002). This was done by selecting “Articles” and “Review” in the side bar tools of the WoS database (Moed, 2002; Bliziotis et al., 2005; Falagas et al., 2006; Cañas-Guerrero et al., 2013). Two persons retrieved and review the articles selected separately based on the criteria aforementioned. All documents were harmonized and agreed upon by the two reviewers for inclusion in the study. Articles that primarily deal with studies on biological and environmental matrices such as water, sediments, soil, human fluid (milk, blood, urine etc.) placenta, air as well as fluid and tissues of aquatic, marine and terrestrial organisms etc., were downloaded in the bibtex file format for the analysis. Retrieved data were analysed for bibliometric (descriptive) statistics, yearly number of publications, countries outputs, journal sources, collaboration networks, and other citation metrics using Rstudio v.3.4.1 software (2017-06-30) with bibliometrix R-package (http://www.bibliometrix.org) (Aria and Cuccurullo, 2017). Bibliometric networks and bibliographic coupling including author, author keyword, citation, co-citation, collaboration, country, co-word analysis, keyword plus networks, and keyword co-occurrences were visualized with the bibliometrix r-package. Other bibliometric indicators such as affiliation were determined using WoS analytic tools. Journal impact factor (IF) was determined using Journal Citation Report (https://jcr.incites.thomsonreuters.com).

## 3 Results and discussion

### 3.1 International evolution of pollutant class – PBDEs

**Fig. 1:**
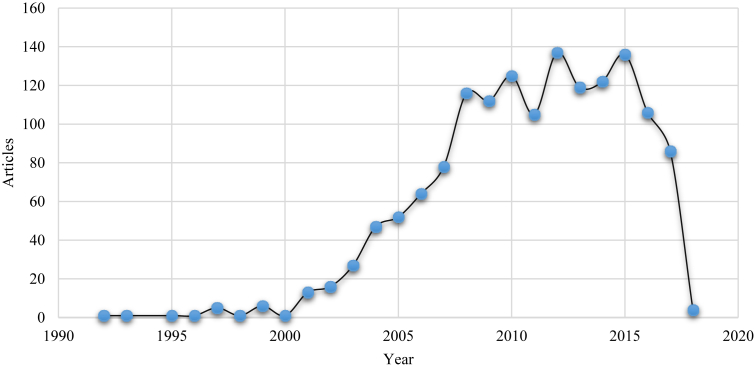
The WoS publications on the analysis of PBDEs from 1992 to 2018.

With the objective of ascertaining the international evolution of this class of pollutants, a broad range of study was carried out. A total of 1482 original articles and reviews were published on the class of pollutants. Production of articles was stable in the first years of this study (1991–1996) with a slight increase in 1997 and 1999. A high increase in publications was noticed in 2006. Production of articles increased tremendously between 2008 and 2015 specifically in 2012 where the highest number of publication of 137 was recorded (Fig. 1 and Table S1). A decline of published articles was recorded in 2016 and 2017 with 106 and 86 articles respectively. Generally, the annual percentage growth from 1992 to 2018 stood at 5.70%.

The steady increase in articles published in this class of pollutant between 2000 and 2010 may be attributed to the ban placed by the European Union (EU), states in the US and Canada on the usage of this pollutant class within these years. This may suggest that there was more research output on this pollutant class within this era especially within the European countries and the US states. However, a sudden decline in articles published on the WoS on this pollutant class may indicate that fewer studies have been conducted on this pollutant class during the ‘post-ban era’.

The penta- and octa- formulate of this pollutant class was banned in some states in the US in the early 2000s (Abbasi et al., 2015), while the European Union banned the use of mixtures and formulations consisting of more than 0.1% (Abbasi et al., 2015). Voluntarily, some U.S manufacturing industries phased out the production of these chemicals due to the potential health threat posed by these chemicals (Gewurtz et al., 2011; Ma et al., 2013b; Route et al., 2014). Furthermore, the European Union banned the production of these chemicals in 2004. Other regulatory restrictions include US importation and usage in 2005 (Route et al., 2014) and the Canadian ban in 2008 (Environment Canada, 2011). However, extensive restriction on the use of deca-BDE has only been done more recently. This congener of BDE is very persistent and more bioaccumulative (U.S. Department of Health and Human Services, 2004). Traceable levels have been found in polar bears in the polar region (Verreault et al., 2005), foxes and birds of prey as well as animals at the bottom the food chain (Voorspoels et al., 2006; Chen et al., 2007). It can also be traced to breast milk (Zhu et al., 2009; Park et al., 2011). Even though it is not immediately toxic, it has the potential to have a long-term adverse effect on foetal development (Li et al., 2014; Lopez-Espinosa et al., 2015). On this account, it was classified to be more toxic than penta- and octa-BDE which have already been banned (Ji et al., 2017). The proposal of Norway to add deca-BDE to the Stockholm convention in 2013 is still under process (Stockholm Convention, 2009). Canada has been assured by major American manufacturers to stop the export of deca-BDE to Canada by 2013 (Environment Canada, 2011). The state of Minnesota passed a ban on four flame retardants (deca-BDE inclusive). This particular congener of PBDE (deca-BDE) was specifically exempted from the Restriction of Hazardous Substances Directive (RoHS) on 15 October 2005. The European Commission took the decision based on the conclusion of the deca-BDE risk assessments on human health and the environment. More so, legal proceedings were launched by the European Parliament and Denmark in January 2006 against the European Commission for exempting deca-BDE from the RoHS Directive. The European Court of Justice annulled the commission decision on 1 April 2008 on the reason that procedural errors were made when establishing the exemption. Therefore, a decision was made on July 2008 by the European Court of Justice that the usage of deca-BDE must be discontinued in electronics and electrical applications (European Court of Justice, 2008; Gewurtz et al., 2011; Chen et al., 2012; Abbasi et al., 2015). In spite of the ban in production and usage of PBDEs in various regions of the world, these pollutant class are still detected in various environmental matrices around the world. Levels have been detected in several food products, aquatic and terrestrial animals (Boon et al., 2002; Voorspoels et al., 2007; Mariussen et al., 2008; Yu et al., 2011; Ma et al., 2013a). Bioaccumulation of PBDEs occurs in the soil and this contaminant shows potential of being bio-magnified (Zou et al., 2007; Kelly et al., 2008; Liang et al., 2010). Reports have shown that these pollutant class and other brominated flame retardants originated majorly from Asia (Suzuki et al., 2006), North America (Batterman et al., 2009) as well as Europe (Mandalakis et al., 2008). Levels of PBDEs in Europe has been found to be lower than those from America (Sjödin et al., 2003). Studies have highlighted that the occurrence of PBDEs in Africa are scanty (Odusanya et al., 2009; Olukunle et al., 2012; Daso et al., 2015).

It is also significant to study the collaboration index in addition to the number of single authors and multiple-authored articles. These data might help to provide valuable information on the scope and consequences of this class of pollutants. The International collaboration index, which represent the number of authorship per articles was 2.73. This indicates that the average number of authors who authorized the articles is approximately 3, which means that there is a high trend of co-authorship (Siamaki et al., 2014). The average citation per article which was 35.7 shows that the distribution of citations to both individual researchers and journal articles are positively skewed (Allison and Stewart, 1974; Price, 1976; Bott and Hargens, 1991).

A total of 4005 authors was observed in all the published articles with 3990 of them written by multiple authors while the remaining 15 were single-authored articles. The average articles per authors and authors per article were 0.37 and 2.7 respectively (Table 1).

**Table 1:** Summarized data on global analysis of PBDEs retrieved from published journals from the WoS.

Descriptions	Counts
Articles	1482
Journal source	226
Keyword Plus (ID), K_p_	2648
Author's Keywords (DE), A_k_	2608
Period	1992–1/02/2018
Average citations per article, A_c/a_	35.7
Authors, A_u_	4005
Author appearances, A_a_	8377
Authors of single authored articles, A_saa_	15
Authors of multiple authored articles, A_mau_	3990
Articles per author, A_/au_	0.37
Authors per article, A_/ar_	2.7
Co-authors per article, C_a/ar_	5.65
Collaboration Index, C_i_	2.73

**Fig. 2:**
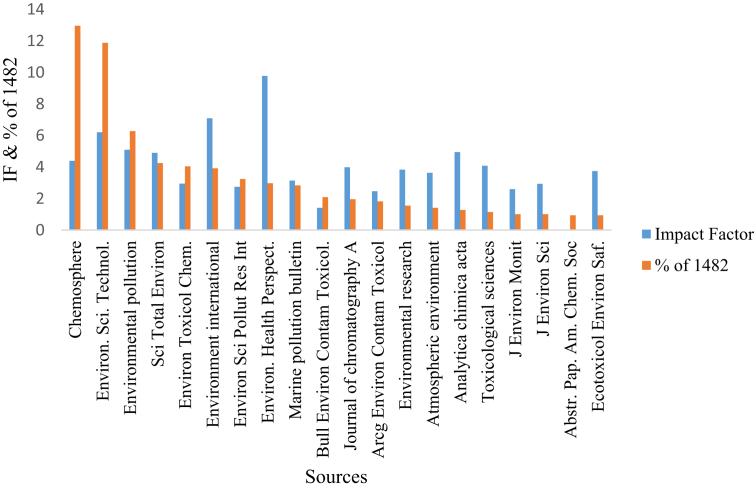
Most relevant sources of article publication on PBDEs and their respective impact factor.

**Fig. 3:**
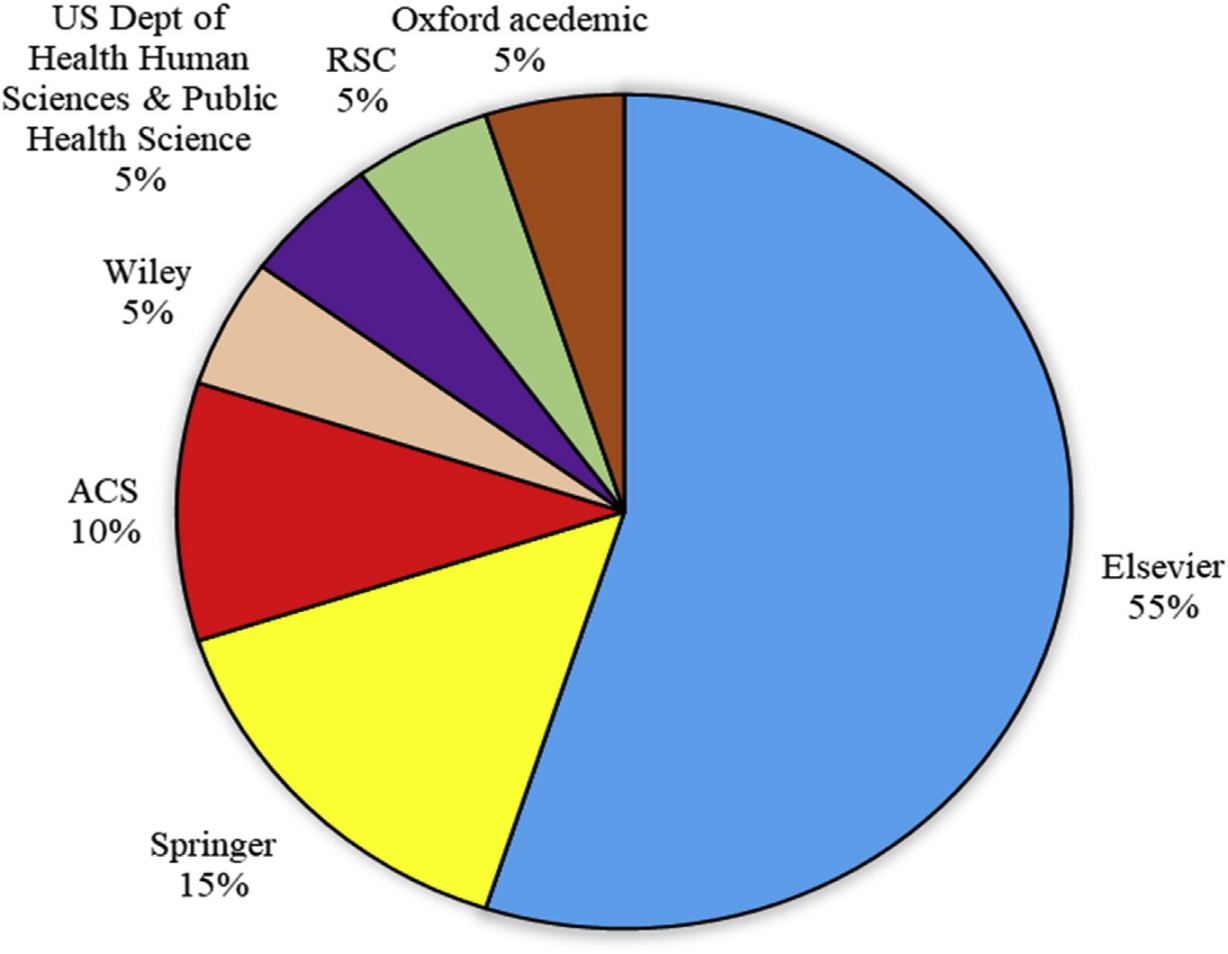
Publishers of the top most 20 journals on the analysis of PBDEs from 1992 to 2018.

Another important aspect of the data was the varieties of the journal house that published articles on these pollutants, taking into account the current year impact factor and the number of citations received per article. The data reveals that Elsevier was responsible for publishing 55% on the 20 most relevant sources on the pollutants with *“Chemosphere”* recording the highest publication (n = 192), followed by *Environmental Pollution* (n = 93) and *Science of the Total Environment* (n = 63) (Figs. 2 and 3 and Table S2). Other publishers that made the top 20 relevant sources include *Springer* (15%), *ACS* (10%) *Wiley* (5%), *US Dept of Health Human Sciences & Public Health Sciences* (5%), *RSC* (5%) and *Oxford Academic* (5%).

### 3.2 Global evolution of the most valuable research topic on PBDEs

**Fig. 4:**
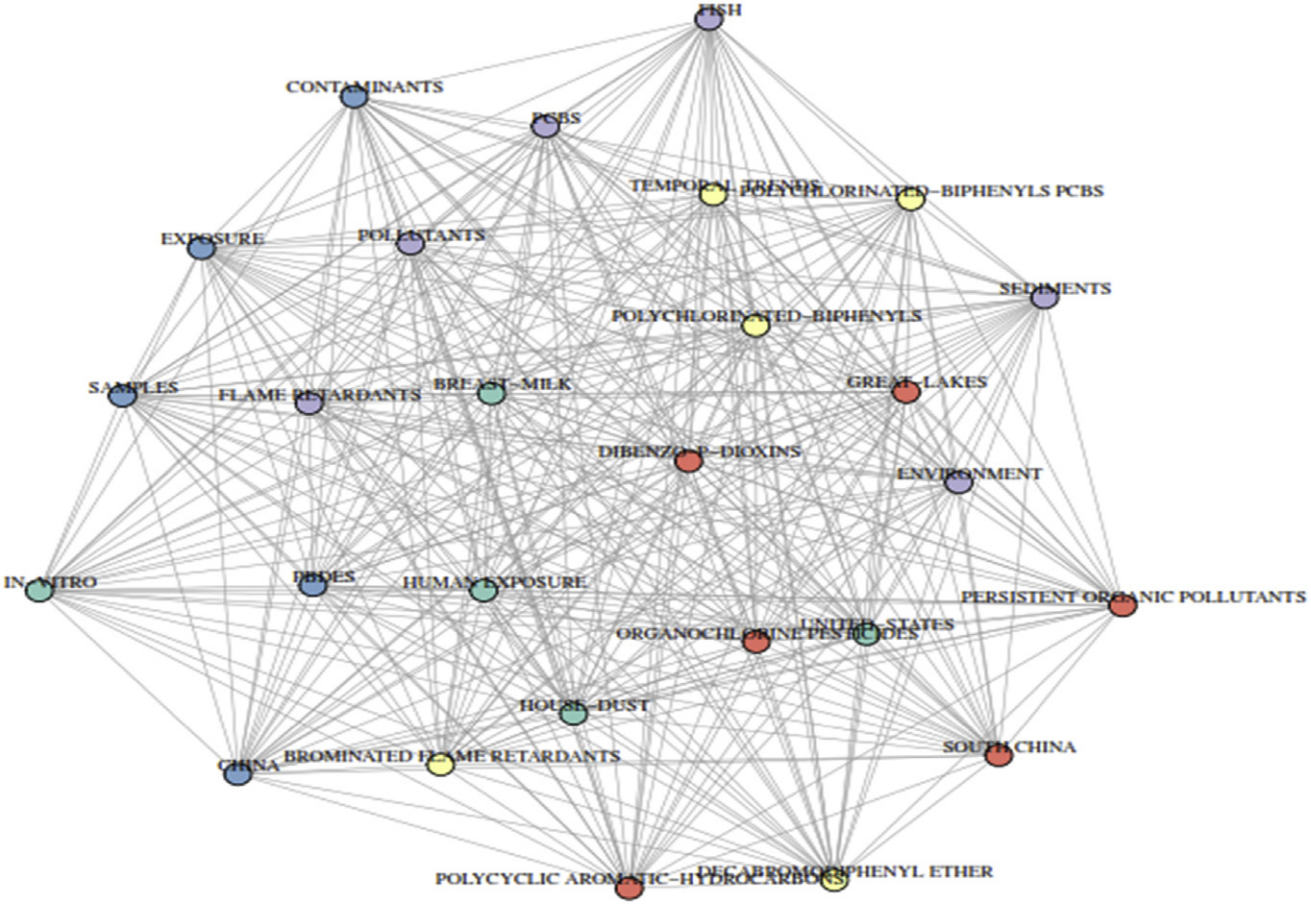
Co-occurrence network of topmost terms associated with global analysis of PBDEs retrieved from the WoS database from 1992 – 2018. Each coloured node in the network represents different term. The node's diameter indicts accompany strength of the term's frequencies of occurrence with others. The lines showed co-occurrence pathway between terms.

Statistical analysis of keyword gives an advantage in the search for discovering the path of science (Zhang et al., 2010). The use of author keywords provides information on the research trends of a particular study as viewed by the researcher. Bibliometric analysis as regards to author keywords can only be found in recent times (Chiu and Ho, 2007) and the usage of author keywords to analyse research trend is still less frequent (Ho, 2007). In this study, keywords plus were used to substantially augment for author keywords. These are additional terms that are frequently seen in the title of the article reference's but do not show up in the title of the article itself. Based on analysis of the keywords associated with PBDEs' class of pollutants, we understand that various organic pollutants like PCBs, OCPs have been analysed together with these pollutants of interest in different environmental matrices when searching the databases of the WoS. To have a broad understanding and collection of keywords, we studied the frequency of the keywords polybrominated diphenyl ethers, PBDEs, polybrominated diphenyl ethers (PBDEs) and PBDE and its associates. Keywords that focused on the evaluation and investigation of PBDEs on sediments, fish, soil, breast milk, e-waste and dust had a percentage occurrence of 3.37%, 3.24%, 2.43%, 2.23%, 1.96% and 1.82% respectively. *“China”* which ranked 1^st^ on countries based on productivity appeared on the 17^th^ position occurring in 30 articles. Other keywords that made the top 20 include flame retardants (3.71%), PCBs (3.58%), exposure (2.77%) amongst others. The analysis of keywords-plus revealed that brominated flame retardants were ranked 1st with a percentage occurrence of 56.72% (n = 841) while POPs was well represented in the top 20 with polychlorinated-biphenyl, dibenzo-p-dioxin and organochlorine pesticides occupying the 2^nd^ (n = 438), 13^th^ (n = 116) and 15^th^ (n = 108) respectively (Fig. 4). This indicates the interrelationship between these pollutants and other organic contaminants (Table 2).

**Table 2:** Most used keywords between 1992 and 2018 retrieved from journals list in the WoS database analysing “PBDEs”.

Rank	N_AK_ (DE)	N_A_ (% of 1482)	N_KP_	Articles (% of 1482)
1	Polybrominated diphenyl ethers	369 (24.90)	Brominated flame retardants	841 (56.75)
2	PBDEs	268 (18.08)	Polychlorinated-biphenyls	438 (29.55)
3	Polybrominated diphenyl ethers (PBDEs)	116 (7.83)	PBDEs	432 (29.15)
4	PBDE	95 (6.41)	Exposure	237 (15.99)
5	Brominated flame retardants	70 (4.72)	Environment	233 (15.72)
6	Flame retardants	55 (3.71)	Decabromodiphenyl ether	175 (11.81)
7	PCBs	53 (3.58)	Flame retardants	159 (10.73)
8	Sediment	50 (3.37)	Persistent organic pollutants	145 (9.78)
9	Fish	48 (3.24)	Human exposure	140 (9.45)
10	Polychlorinated biphenyls	44 (2.97)	South china	136 (9.18)
11	Exposure	41 (2.77)	Breast-milk	134 (9.04)
12	Polybrominated diphenyl ether	40 (2.70)	Polychlorinated-biphenyls PCBs	120 (8.10)
13	Bioaccumulation	38 (2.56)	Dibenzo-p-dioxins	116 (7.83)
14	Human exposure	36 (2.43)	Fish	113 (7.62)
15	Soil	36 (2.43)	Organochlorine pesticides	108 (7.29)
16	Breast milk	33 (2.23)	Great-lakes	104 (7.02)
17	China	30 (2.02)	Pollutants	98 (6.61)
18	Persistent organic pollutants	30 (2.02)	Samples	98 (6.61)
19	E-waste	29 (1.96)	PCBs	95 (6.41)
20	Dust	27 (1.82)	China	92 (6.21)

*N*_*AK*_ number of author's keywords, *N*_*A*_ number of articles, *N*_*KP*_ number of keywords-plus.

**Fig. 5:**
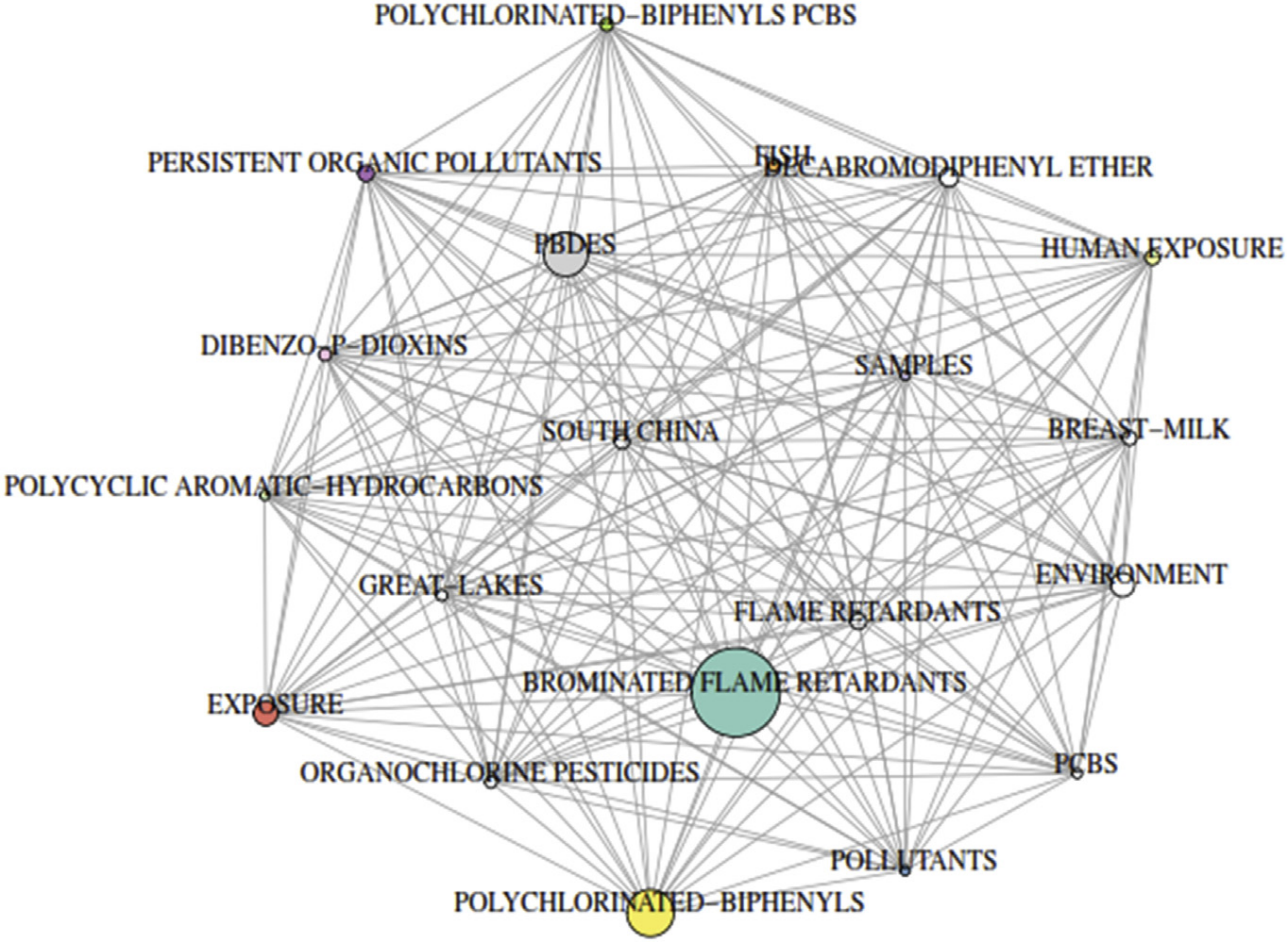
Keyword co-occurrence network on global analysis of PBDEs retrieved from the WoS database from 1992 – 2018. Each coloured node in the network represents different term. The node's diameter indicts accompany strength of the term's frequencies of occurrence with others. The lines shows co-occurrence pathway between terms.

Fig. 4 gives co-occurrence network of the topmost terms associated with PBDEs. Keywords' co-occurrences network of PBDEs is also represented in Fig. 5. These terms include brominated flame retardants, polychlorinated-biphenyls, PBDEs, exposure, environment, decabromodiphenyl ether, flame retardants, persistent organic pollutants, human exposure, South China amongst others.

### 3.3 Countries based productivity and citations on PBDEs

In studying the countries that were most productive in analysing PBDEs class of pollutants in biological and environmental matrices between 1992 and 2018, quantitative and qualitative parameters (countries, number of articles, frequency, average article citations and total average citations) were used. Table 3 shows that 20 countries were most productive in studying the pollutants accounting for over 90% of the published articles. It is worth noting that the Asia, European and America countries dominated the 20 most productive countries on the analysis of PBDEs. This might be attributed to economic advancement, availability of funds, the presence of sophisticated analytical tools and international collaboration within developed countries. The only African country that was present in this category was South Africa, occupying the 17^th^ position. This can be positively correlated with the result obtained from the biennial global inter-laboratory assessment which showed a very low involvement of Africa countries on the analysis of POPs in the continent (http://www.unep.org/chemicalsandwaste/Science/tabid/268/Default.aspx). Over the years, some chemical societies such as American Chemical Society and Royal Society of Chemistry have been involved in capacity building and training African emerging researchers on the instrumental analysis on POPs, however, more societies should get involved in order to bridge this gap.

**Table 3:** Countries based productivity and citations on PBDEs from articles from 1992 to 2018.

Rank	M_PA_					T_C/C_		
	Country	Articles	% of 1482	Freq	Rank	Country	T_C_	A_AC_
1	China	445	30.03	0.30	1	USA	14784	46.06
2	USA	321	21.66	0.22	2	China	10822	24.32
3	Canada	105	7.09	0.07	3	Sweden	5047	77.65
4	Sweden	65	4.39	0.04	4	Canada	4416	42.06
5	Japan	52	3.51	0.04	5	Spain	2195	42.21
6	Spain	52	3.51	0.04	6	Japan	2006	38.58
7	Korea	39	2.63	0.03	7	England	1807	69.5
8	Taiwan	34	2.29	0.02	8	Belgium	1533	56.78
9	Italy	28	1.89	0.02	9	Norway	1007	50.35
10	Belgium	27	1.82	0.02	10	Netherlands	973	69.5
11	England	26	1.75	0.02	11	Korea	862	22.1
12	Germany	23	1.55	0.02	12	Germany	807	35.09
13	Australia	21	1.42	0.01	13	Italy	765	27.32
14	Norway	20	1.35	0.01	14	Denmark	747	41.5
15	Denmark	18	1.21	0.01	15	Taiwan	738	21.71
16	France	18	1.21	0.01	16	Australia	722	34.38
17	Poland	17	1.15	0.01	17	Singapore	477	47.7
18	South Africa	17	1.15	0.01	18	France	338	18.78
19	Netherlands	14	0.94	0.01	19	Kuwait	294	26.73
20	Greece	11	0.74	0.01	20	Turkey	266	38

*M*_*PA*_ Most productive countries, *T*_*C/C*_ Total citations per country, *A*_*AC*_ Average Article Citations, *T*_*C*_ Total Citations.

The People's Republic of China was ranked first on countries based on productivity with a total of 445 published articles, accounting for 30% of the total articles published as shown in Table 3. While USA and Canada were ranked second and third with 321 (21.7%) and 105 (7.1%) articles respectively. The position of the People's Republic of China specifically in the number one position may be ascribed to their huge spending on research and development (R&D) and their enthusiasm on scientific knowledge in industries which makes them responsible for 20% expenditure on R&D globally (Veugelers, 2017). Denmark and France were tied on the 15^th^ position with 18 articles publications. Countries lagging far behind in the top 20 are Netherland and Greece occupying the 19th and 20th position with 14 and 11 articles respectively. Publication frequency among countries was within the range of 0.01–0.30. Other parameters of good quality observed in Table 3 are the total citations (T_C_) and average article citations (A_AC_) (grouped as total citation per country T_C/C_). In the first of these, USA was ranked first with a total citation of 14784 recording an average article citation of 46.06, while the People's Republic of China occupied the second position with a total citation of 10822 (A_AC_ 24.32). The dominance of China and USA, exchanging the first and second position in the category of ‘M_PA_’ and ‘T_C/C_’ reveals the high international diffusion of their articles (Cañas-Guerrero et al., 2013). The only country that retained its position in these two categories was Germany in the 12^th^ position. Although the analysis of PBDEs may be insignificant in the list of scientific publications, it gives an idea of the competition between China and USA in scientific technologies globally. In general, other cutting-edge areas that have experienced significant expansion in China between 1996 and 2005 are nanotechnologies and energetic materials (Kostoff et al., 2007). In comparison to the USA, the main focuses of articles from China were physical and engineering science while the USA dominated in the areas including psychological, medical and social sciences. A significant expansion has been noticed in China's publications between 1996 and 2005 (Kostoff et al., 2007, 2008). Furthermore, following the approval of US$43 million for POPs project in some Asian countries by the global environment facility (IISD, 2013), it is expected that more studies will be carried out in that part of the world, thereby increasing the number of publications. Other programs which will also foster the analysis of PBDEs in developing countries include the UN Development Programme on the reduction of PBDEs from bad management practice in Indonesia and UNIDO project for reduction of POPs in Senegal (http://sdg.iisd.org/news/gef-approves-over-us43-million-for-12-pops-projects/).

Asian countries which made it to the top 20 include Japan, Korea, Taiwan, Singapore and Kuwait with a total citation per country of 2006, 862, 738, 477 and 294 respectively. Turkey occupied the 20^th^ position with a total citations average of 266. No African country was included in this category (Table 3).

The dominance of Asia, European and America countries in these categories (Table 3) may also be attributed to the fact that these three continents lead in the usage of brominated flame retardants compared to other continents of the world (Table 4) (Law et al., 2006). Besides, major companies producing brominated flame retardants are domiciled in these continents. These companies include Ethyl Corporation, now known as Albermarle Corporation based in the US, Great Lakes Chemical Corporation (US and United Kingdom), ICL-IP Europe BV previously known as Eurobrom (Netherlands) and Dead Sea Bromine Company Limited (Israel). Other companies are Riedel de Haen Chemicals (Hoechst Group), Atochem (France), Abermarle Chemicals (Belgium), Potasse et Produit Chimiques (Rhone Poulene Group), as well as Nippo, Matsunaga and Tosoh (Japan) (WHO/ICPS, 1994; De Wit, 2002). In 1992, global production of all forms of brominated flame retardants was estimated to be 150000 metric tons/year (De Wit, 2002). Twenty-five percent of this distribution was to Europe, 30% to the Far East and 40% to North America (Arias, 1992; KEMI, 1994). Furthermore, global production of penta-, octa and deca BDE technical mixtures was estimated to be 4000, 6000 and 3000 metric tons/year respectively (Arias, 1992). Usage of PBDEs in the European Union in 1999 was approximately 150 metric tons for penta formulations, 400 and 7000 metric tons for octa and deca BDE formulation respectively (De Poortere, 2000).

**Table 4:** Global usage of brominated flame retardants (tonnes) in different continents of the world in 2001 (Law et al., 2006).

	TBBP-A	HBCD	Deca-BDE mixture	Octa-BDE mixture	Penta-BDE mixture	Total
Americas^a^	18000	2800	24500	1500	7100	53900
Europe	11600	9500	7600	610	150	29460
Asia	89400	3900	23000	1500	150	117950
Other continents	600	500	1050	180	100	2430
Total	119700	16700	56100	3790	7500	203790
% Global usage	59	8	27	2	4	100

a All America continent but North America in particular where USA is the major user.

**Fig. 6:**
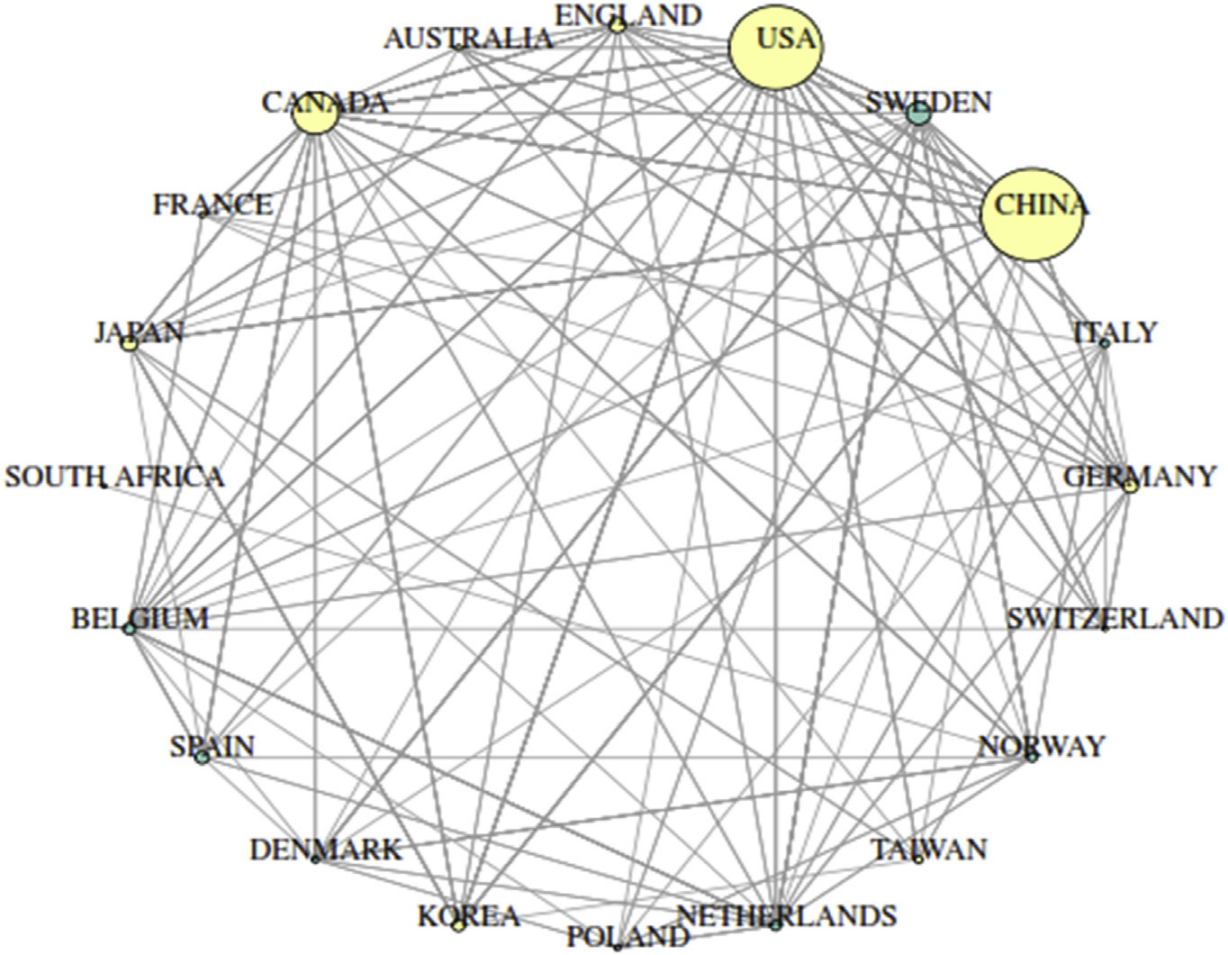
Twenty topmost country collaboration network on the analysis of PBDEs retrieved from the WoS database from 1992 to 2018. Each coloured node in the network represents different country and the node's diameter indict accompany strength of the country's collaboration with other countries. The lines showed collaboration pathway between countries.

Furthermore, Fig. 6 revealed the countries with the most collaboration networks on the analysis of PBDEs. China retained the top spot on the list of countries with the most collaboration network followed by the USA, Canada and Sweden. However, other countries with high collaborative strengths include Germany, Netherlands, Korea, Denmark, Spain, Belgium, Japan and France with pathways that ranged from 6 to 12 collaborations within countries. The only African country found in this category was South Africa with a single collaboration with Norway. In this collaboration, eggs samples from different species of birds were collected in South Africa, frozen at −20 °C, wrapped in an aluminium foil and transported to Norway for GC-MS analysis (Polder et al., 2008). The high number of research publications from European countries may be attributed to the existence of breast milk monitoring programs of POPs in countries like Sweden, Netherland and Germany (Hooper and McDonald, 2000).

### 3.4 Research evolution by institution

Table 5 showed that most of the productive researchers on PBDEs analysis are domiciled in Asian countries as most authors' institutions are located in the People's Republic of China. This may be attributed to the fact that Asian countries lead in the global usage of brominated flame retardants and China specifically still produces and uses enormous quantities of commercial PBDEs (Chen et al., 2012) hence attracting more study by researchers within the country, compared to the EU and US where restrictions and regulations have been placed on the usage of PBDEs (penta-, octa- and deca-) in the early 2000s. In China, Electronic Information Products Pollution Control Measures is the only regulation related to PBDEs (Chen et al., 2012) with its stipulated limit of 1000 ppm PBDE content in electrical appliances. In addition to this regulation, China has joined the Stockholm Convention on POPs and in July 2007, the plan for the implementation of Stockholm Convention includes the phase-out of commercial penta- and octa-BDE before the end of the year 2030 (Wei et al., 2007; Ma et al., 2012; Wang et al., 2012).

**Table 5:** Twenty most productive researchers on PBDEs analysis retrieved 1992 to 2018 from WoS Database.

Rank	Authors	Research Institutions	ORCID ID	Country	Articles	g index	h index	m index	TC	NP
1	Wang, Y	SKLECE	NA	China	54	33	19	1.73	1132	50
2	Li, J	China National Centre of Food Safety Risk Assessment, Beijing	NA	China	41	32	20	1.82	1059	41
3	Li, Y	Basic Medicine, Ningbo University	NA	China	40	25	16	1.45	752	45
4	Mai, B	GIGCAS	0000-0001-6358-8698	China	37	30	20	1.82	1276	30
5	Fu, J	School of Environment and Chemical Engineering, Shanghai University, Shanghai	NA	China	35	25	15	1.36	652	29
6	Jiang, G	SKLECE	NA	China	32	22	14	1.27	577	22
7	Kannan, K	School of Public Health, State University of New York	NA	USA	32	22	14	1.27	839	22
8	Zhang, X	GIGCAS	NA	China	32	32	18	1.64	1081	34
9	Zhang, Y	GIGCAS	NA	China	31	26	11	1.00	731	26
10	Covaci, A	Toxicological Center, University of Antwerp	0000-0003-0527-1136	Belgium	27	17	13	1.18	679	17
11	Wang, X	Institute of Tibetan Plateau, CAS		China	27	23	14	1.27	561	25
12	Bergman, A	Department of Environmental Chemistry, Stockholm University, Stockholm	0000-0003-3403-093X	Sweden	25	13	11	1.00	720	13
13	Luo, X	GIGCAS	0000-0002-2572-8108	China	25	20	16	1.45	970	20
14	Sheng, G	GIGCAS.			25	19	11	1.00	497	19
15	Chen, L	GIGCAS		China	24	20	12	1.09	413	22
16	Letcher, RJ	Government of Canada, University of Windsor, Universiteit Utrecht, Carleton University, University of Toronto Scarborough	0000-0002-8232-8565	Canada	24	16	11	1.00	787	16
17	Li, X	The Hong Kong Polytechnic University	NA	Hong-Kong	24	24	12	1.09	595	26
18	Chen, J	School of Chemistry and Environment, South China Normal University, Guangzhou.	NA	China	22	18	9	1.00	336	21
19	Chen, S	GIGCAS	NA	China	22	25	18	1.64	1084	25
20	Wu, Y	School of Public Health, Capital Medical University, Beijing.	NA	China	22	22	13	1.18	500	24

*GIGCAS* Guangzhou institute of geochemistry Chinese academy of sciences, Guangzhou; *SKLECE* state key laboratory of environmental chemistry and ecotoxicology Beijing; *TC* Total citation, *NP* number of publication, *NA* not available.

**Fig. 7:**
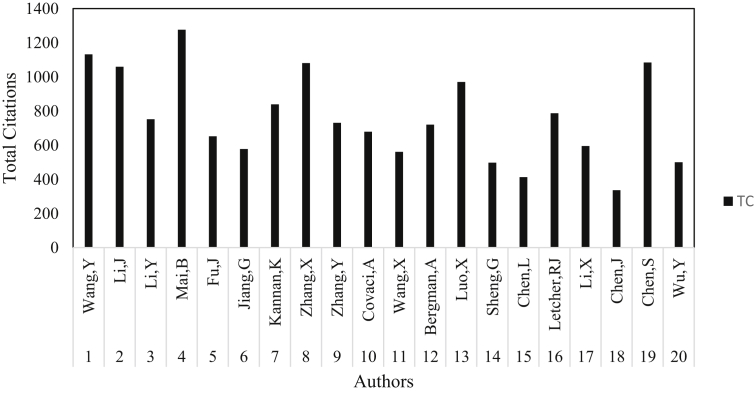
Most cited authors on the analysis of PBDEs retrieved from the WoS database from 1992 to 2018.

Wang, Y. affiliated with State Key Laboratory of Environmental Chemistry and Ecotoxicology Beijing occupied the first spot with 54 articles. Another researcher from this institution was Jiang, G with a total article of 32 occupying the sixth position. The only three non-Asians scientists which made it to the top twenty most productive researchers on PBDEs were Kannan K., Covaci, A., Bergamn, A from USA, Belgium and Sweden respectively. This shows that China is gradually gaining more ground in overtaken the world superpowers (US and EU) on the leadership of science and technology (Shelton and Foland, 2009) as shown in their numerous numbers of scientific publications. Since the release of the National Implementation Plan in July, 2007 by the Chinese government for the implementation of the Stockholm Convention on POPs (www.gov.cn), more studies have been carried out on PBDEs and other POPs and their elimination in the ecosystem (Shi et al., 2005; Yang et al., 2013). Locations in China that have received more attention on the analysis of PBDEs as pollutants include the Pearl River (Mai et al., 2005; Zhang et al., 2009; Chen et al., 2011), Bohai Sea and Yellow Sea (Liu et al., 2011a, b). More so, Mai. B, Chen. S, Zhang. X, and Luo. X stand out as the most cited authors on the analysis of PBDEs class of pollutants with a total citation of 1276, 1084, 1081, 970 respectively (Fig. 7).

Table 6 presents the most cited manuscript on the pollutants. From the result, an article published by Rudel et al. (2003) on analysis of POPs in indoor air and dust topped the ranking of the most cited study with an average total citation of 36 times per year. In this article, indoor air and dust samples from 120 homes were analysed for seven classes of POPs (alkylphenols & alkyl phenol ethoxylates, phthalates, parabens, polyaromatic hydrocarbons, PBDEs, pesticides and phenols). The sample matrices, the number of samples and varieties of POPs analysed may be responsible for the significant number of citation (540). Furthermore, 55% of the articles in the top 20 most cited manuscripts were financially supported by agencies and institutes. Most of the journals in the category were published by *Environmental Science and Technology.*

**Table 6:** Twenty most cited manuscript on PBDEs analysis retrieved from 1992 to 2018 from the WoS Database.

Rank	Title	Authors/Year	Journal	Financial support	TC	TC/Year
1	‘Phthalates, Alkylphenols, Pesticides, Polybrominated Diphenyl Ethers, and Other Endocrine-Disrupting Compounds in Indoor Air and Dust’.	(Rudel et al., 2003)	*Environ. Sci. Technol.*	Massachusetts Department of Public Health	540	36
2	‘Distribution of Polybrominated Diphenyl Ethers in Sediments of the Pearl River Delta and Adjacent South China’.	(Mai et al., 2005)	*Environ. Sci. Technol.*	National Basic Research Program of China, the Chinese Academy of Sciences Natural Science Foundation	376	28.9
3	‘Polybrominated diphenyl ethers (PBDEs) in US mothers' milk’.	(Schecter et al., 2003)	*Environmental Health Perspect.*	Not stated	354	23.6
4	‘Exponential increases of the brominated flame retardants, polybrominated diphenyl ethers, in the Canadian Arctic from 1981 to 2000’.	(Ikonomou et al., 2002)	*Environmental Health Perspect.*	Not stated	350	21.9
5	‘Polybrominated diphenyl ether flame retardants in the North American environment’.	(Hale et al., 2003)	*Environ. Int.*	Not stated	339	22.6
6	‘Spatial distribution of polybrominated diphenyl ethers and polychlorinated dibenzo-p-dioxins and dibenzofurans in soil and combusted residue at Guiyu, an electronic waste recycling site in southeast China’.	(Leung et al., 2007)	*Environ. Sci. Technol.*	The Research Grants Council of the University Grants Committee of Hong Kong	334	30.4
7	‘Effects of short-term in vivo exposure to polybrominated diphenyl ethers on thyroid hormones and hepatic enzyme activities in weanling rats’.	(Zhou et al., 2001)	*Toxicol. Sci.*	U.S. EPA/UNC Toxicology Research Program	331	19.5
8	‘Analysis of polybrominated diphenyl ethers in Swedish human milk. A time-related trend study, 1972–1997’.	(Meironyté et al., 1999)	*J. Toxicol. Health (Part A).*	Not stated	331	17.4
9	‘Polybrominated diphenyl ethers in house dust and clothes dryer lint’.	(Stapleton et al., 2005)	*Environ. Sci. Technol.*	National Institute of Standards and Technology's National Research Council Postdoctoral Research program.	325	25
10	‘Polybrominated diphenyl ethers in maternal and fetal blood samples’.	(Mazdai et al., 2003)	*Environmental Health Perspect.*	Not stated	308	20.5
11	‘Exposure of Americans to polybrominated diphenyl ethers’.	(Lorber, 2008)	*J. Expo. Sci. Environ. Epidemiol.*	Not stated	299	29.9
12	‘Polybrominated diphenyl ethers and hexabromocyclododecane in sediment and fish from a Swedish river’.	(Sellström et al., 1998)	*Environ Toxicol Chem*	Swedish Environmental Protection Agency	289	14.4
13	‘Flame retardant exposure: polybrominated diphenyl ethers in blood from Swedish workers’.	(Sjödin et al., 1999)	*Environmental Health Perspect.*	Not stated	288	15.2
14	‘Effects of polybrominated diphenyl ethers (PBDEs) and polychlorinated biphenyls (PCBs) on thyroid hormone and vitamin A levels in rats and mice’.	(Hallgren et al., 2001)	*Arch. Toxicol.*	Swedish Environmental Protection Agency and Foundation for strategic environmental research	279	16.4
15	‘Polybrominated diphenyl ethers in indoor dust in Ottawa, Canada: implications for sources and exposure’.	(Wilford et al., 2005)	*Environ. Sci. Technol.*	Natural Environment Research Council and Corus U.K	271	20.8
16	‘Levels of polybrominated diphenyl ether (PBDE) flame retardants in animals representing different trophic levels of the North Sea food web’.	(Boon et al., 2002)	*Environ. Sci. Technol.*	Bromine Science and Environmental Forum (BSEF), Brussels, Belgium	267	16.7
17	‘Concentrations and spatial variations of polybrominated diphenyl ethers and other organohalogen compounds in Great Lakes air’.	(Strandberg et al., 2001)	*Environ. Sci. Technol.*	Not stated	266	15.6
18	‘Concentrations of polychlorinated biphenyls in indoor air and polybrominated diphenyl ethers in indoor air and dust in Birmingham, United Kingdom: implications for human exposure’.	(Harrad et al., 2006)	*Environ. Sci. Technol.*	Not stated	256	21.3
19	‘Identification and quantification of polybrominated diphenyl ethers and methoxy-polybrominated diphenyl ethers in Baltic biota’.	(Haglund et al., 1998)	*Environ. Sci. Technol.*	Swedish Environmental Protection Agency “Persistent Organic Pollutants” scientific program	251	12
20	‘Polybrominated diphenyl ether (PBDE) levels in an expanded market basket survey of US food and estimated PBDE dietary intake by age and sex’.	(Schecter et al., 2006)	*Environmental Health Perspect.*	CS Foundation, Warsh Mott Legacy	245	20.4

*TC* total citation.

### 3.5 Production, technical use and recycling pathways of PBDEs in regions cited in bibliometric indices

Over the years, three congeners of PBDEs (penta-, octa- and deca-BDE) have been produced in developed regions of the world. However, there is paucity of information on the productions of these pollutant class in developing countries (BSEF, 2006). Historical production of all PBDEs was estimated to be 1.3 million and 1.5 million tonnes from 1970 to 2005 according to data collated for POPs reviewing committee of the Stockholm Convention (UNEP, 2010). Of all three congeners of PBDEs, commercial penta-BDE was produced in China, Japan, USA and EU. Its production was stopped in the EU in 2004. It is assumed that the production of this congener has declined in proportion since the beginning of the millennium (UNEP, 2010). Also, commercial octa-BDE which was produced in the UK, Netherlands, Israel, France and Japan was stopped from production in the EU and USA in 2004. Deca-BDE, which was not initially included in the list of POPs recorded an estimated production of 1.1 million tonnes until 2005. While the production of other BDE congeners (octa- and penta-) ended in 2004, production of deca-BDE was still ongoing (Jinhui et al., 2017). It was reported by the US Toxic Release Inventory that about 31 metric tons of deca-BDE is released to the air in 2003 (ACAP/AMAP, 2007). Some products (textiles, furniture's, electrical and electronic gadgets and building and construction materials) where these contaminants are used as flame retardants are exported out of the country as finish products while others remain in the country. However, due to confidentiality reasons, there are no existing data from the US on the imports and export products (ACAP/AMAP, 2007). Canada, a major recipient of this products estimated that about 6000 metric tons of deca-BDE could be imported as products from the US annually. More so, the consumption of BFRs (per capita basis) in USA and Canada is estimated to be 330 tons/year/million inhabitants and 210 tons/year/million inhabitants respectively. The Scandinavian countries have lower usage of BFRs (de Wit et al., 2010). Sweden have reported limited export and import of deca-BDE, also its usage has been restricted but other brominated flame retardants such as tetrabromobisphenol (TBBPA) is still in use for the production of electrical and electronic appliances. This means that Sweden still exports goods containing TBBPA (ACAP/AMAP, 2007). Wide application on the use of PBDEs has been found in the electrical and electronic sectors especially in Asian countries. The demand for electronics has rapidly increased the consumption of PBDE in Asia countries. Furthermore, export of these products from developed countries in the name of “recycling” to developing countries, especially for South and East Asia (India, Pakistan and China) is a major pathway in which these chemicals get into the environment in these countries (Martin et al., 2004). China, one the world largest manufacturers of electrical appliances incorporate a large amount of PBDEs to meet up with fire regulations. The annual demands of BFRs for products in China were estimated to be 7 × 10^7^ to 8.7 × 10^7^ kg in 2005–2010 (increasing rate – 7% to 8% per year) (Jiang, 2006). Apart from domestic production, a greater portion of e-waste is transported to China for recycling (Chen et al., 2009). E-waste is among the fastest growing source of waste worldwide (Widmer et al., 2005; Lundgren, 2012; Grant et al., 2013).

It was estimated by the United Nations Environmental Program (UNEP) that the amount of e-waste generated in 2012 alone is enough to fill up a hundred Empire state building, averaging to about 15 pounds (6.8 kg) per person (StEP Report, 2013). In 2012, the United State and China reportedly generates about 10 million tons and 11.1 million tons of e-waste respectively (StEP Report, 2013). This implies that each American generates about 29.5 kg of e-waste which is less than 5 kg per person in China. The large amount of e-waste generated globally is problematic but more concerning is the adverse effect of these waste to humans and animals. It was reported that about 145 million tonnes of imported e-waste which contains about 2.61 × 10^8^ kg PBDEs were recycled in South China in 2002 (Martin et al., 2004). During the recycling process, PBDEs in these products may be released and be accumulated in living tissues of animals and humans. In a study carried out by Yuan et al. (2008), Chinese waste workers recorded higher levels of serum PBDEs and thyrotropic hormone compared to levels found in the control groups. Hence, the increased exposure of these pollutants form e-waste recycling may hinder the activities of the thyroid hormones thereby bringing about other adverse health effects. PBDE enhanced acrylonitrile butadiene styrene polymers was predominantly used in casing of electrical and electronic equipment (EEE), particularly for photocopying machines, TV and computer monitors as well as other business printing machines. Disposal of waste generated from electrical and electronic equipment has been a major call for concern in developing countries (UNEP, 2006). The technology used for the management and disposal of e-waste is still poorly developed (Osibanjo and Nnorom, 2007). Developing countries in Africa such as Nigeria and Ghana as well as transition country like South Africa which are recipients of EEE products from developed countries currently experience low-end management activities of e-waste and this is a great call for concern, taking cognisance of the fact that hazardous chemicals like PBDEs are released into the environment (Ahn et al., 2006; Wang et al., 2007; Luo et al., 2009; Akortia et al., 2016). The main source of exposure of PBDEs to the environment is when products containing these pollutants are incinerated, disposed on a dumpsite or used as landfills (Chen et al., 2009). Other industries which have to implore its usage are the textiles industries, transportation industries, furniture and carpet industries as well as the recycling industries (SSC et al., 2012). It is estimated that about 90–95% of commercial penta-BDE was used in the treatment of polyurethane foams (Vyzinkarova and Brunner, 2013) majorly for furniture and automotive applications (European Chemicals Bureau, 2000; Sjödin et al., 2003).

## 4 Conclusions

This bibliometric study presents a great involvement on the analysis of this class of pollutants in various environmental and biological matrices around the world. The dominance of research activities on this class of pollutants by China and USA across all indices (authors, centres, and countries) gives an idea of the competition between these two countries on becoming the world power in scientific publication. The results reveals that European countries are lagging behind China and USA across all indices in the analysis of PBDEs. In addition, lack of funds, sophisticated analytical tools, international collaboration and awareness may be the factors responsible for deficiencies in studies on the analysis of PBDEs in developing countries especially in Africa. Hence more studies should be carried out in Africa and European environment in order to counterbalance the dominance of the US and China, bearing in mind that these pollutants takes several years to phase out of the eco-system.

It is further recommended that other organic pollutants that are of environmental and human concern should attract further bibliometric studies; this will assist the government in formulating policies around organic pollutants especially in countries where these pollutants have been extensively used in the past. This will also assist researchers in streamlining their choice of journals and afford them an idea on which to read and where to publish their articles.

Several limitations are associated with our study, in terms of data collection and interpretation. In this study, we used the WoS database to identify journals that are related to this pollutant class. However, articles published in non-WoS-cited journals were not included in this study despite the fact that their content also reflects scientific productivity. This situation is unique to regions with authors who are not English speakers, where researchers publish their findings in local/regional journals of their own language (Rosmarakis et al., 2005). Secondly, not all research on the analysis of this pollutant class is published. The counts of journals do not depict quality. Hence bibliometric tool does not provide subjective criticism in journals analysing these pollutant class particularly in terms of data collection, methodology, result, discussion and conclusions. The third limitation is related to the database used for this study. The WoS whose database has been operational for several decades undergoes some changes over the years especially in the expansion of journal coverage (Bornmann and Mutz, 2015). As this study is majorly based on the analysis of PBDEs in published articles, we assume that a change of database does not alter our search results.

## Declarations

### Author contribution statement

Olisah Chijioke: Conceived and designed the analysis; Analyzed and interpreted the data; Wrote the paper.

Okoh O. Omobola, Okoh I. Anthony: Contributed analysis tools or data; Wrote the paper.

### Funding statement

This work was supported by the South African Medical Research Council, the National Research Foundation (NRF) of South Africa (112780).

### Competing interest statement

The authors declare no conflict of interest.

### Additional information

No additional information is available for this paper.
