# Supplementary material for: A bibliometric analysis of investigations of polybrominated diphenyl ethers (PBDEs) in biological and environmental matrices from 1992 – 2018
Source: Heliyon. 2018 Nov 26;4(11):e00964. doi: 10.1016/j.heliyon.2018.e00964 (PMC6260465; doi:10.1016/j.heliyon.2018.e00964)
Supplement: Supplementary Material [file mmc1.docx]

**A bibliometric analysis of investigations of polybrominated diphenyl ethers (PBDEs) in biological and environmental matrices from 1992 – 2018**

Olisah Chijioke ^1,2,3^ Okoh O. Omobola ^1, 2,3^ and Okoh I. Anthony ^2,3^.

^1^Department of Pure and Applied Chemistry, University of Fort Hare, Alice 5700, South Africa.

^2^Applied and Environmental Microbiology Research Group (AEMREG), Alice 5700, South Africa

^3^SAMRC, Microbial Water Quality Monitoring Centre, University of Fort Hare, Alice 5700.

**Corresponding author**

Olisah Chijioke

Email: [olisah.chijioke@gmail.com](mailto:olisah.chijioke@gmail.com)

Phone number: +27635146813

ORCID identifier 0000-0002-7714-3056

The supporting information consist of

**Table S1.** Annual Scientific Production on global analysis of PBDEs retrieved from the WoS from 1992 to 2018

**Table S2.** Top twenty most relevant sources of article publication on analysis of PBDEs retrieved from the WoS from 1992 to 2018

**Table S1.** Annual Scientific Production on global analysis of PBDEs retrieved from the WoS from 1992 to 2018

| S/N | Year | Number of Articles |
| --- | --- | --- |
| 1 | 1992 | 1 |
| 2 | 1993 | 1 |
| 3 | 1995 | 1 |
| 4 | 1996 | 1 |
| 5 | 1997 | 5 |
| 6 | 1998 | 1 |
| 7 | 1999 | 6 |
| 8 | 2000 | 1 |
| 9 | 2001 | 13 |
| 10 | 2002 | 16 |
| 11 | 2003 | 27 |
| 12 | 2004 | 47 |
| 13 | 2005 | 52 |
| 14 | 2006 | 64 |
| 15 | 2007 | 78 |
| 16 | 2008 | 116 |
| 17 | 2009 | 112 |
| 18 | 2010 | 125 |
| 19 | 2011 | 105 |
| 20 | 2012 | 137 |
| 21 | 2013 | 119 |
| 22 | 2014 | 122 |
| 23 | 2015 | 136 |
| 24 | 2016 | 106 |
| 25 | 2017 | 86 |
| 26 | 2018 | 4 |
| 27 | 2017 | 86 |
| 28 | 2018 | 4 |

**Table S2.** Top twenty most relevant sources of article publication on analysis of PBDEs retrieved from the WoS from 1992 to 2018

| S/N | Sources | Number of articles published | % of 1482 |
| --- | --- | --- | --- |
| 1 | Chemosphere | 192 | 12.955 |
| 2 | Environmental Science & Technology | 176 | 11.876 |
| 3 | Environmental Pollution | 93 | 6.275 |
| 4 | Science of the Total Environment | 63 | 4.251 |
| 5 | Environmental Toxicology and Chemistry | 60 | 4.049 |
| 6 | Environment International | 58 | 3.914 |
| 7 | Environmental Science and Pollution Research | 48 | 3.239 |
| 8 | Environmental Health Perspectives | 44 | 2.969 |
| 9 | Marine Pollution Bulletin | 42 | 2.834 |
| 10 | Bulletin of Environmental Contamination And Toxicology | 31 | 2.092 |
| 11 | Journal of Chromatography A | 29 | 1.957 |
| 12 | Archives of Environmental Contamination and Toxicology | 27 | 1.822 |
| 13 | Environmental Research | 23 | 1.552 |
| 14 | Atmospheric Environment | 21 | 1.417 |
| 15 | Analytica Chimica Acta | 19 | 1.282 |
| 16 | Toxicological Sciences | 17 | 1.147 |
| 17 | Journal of Environmental Monitoring | 15 | 1.012 |
| 18 | Journal of Environmental Sciences | 15 | 1.012 |
| 19 | Abstracts of Papers of the American Chemical Society | 14 | 0.945 |
| 20 | Ecotoxicology and Environmental Safety | 14 | 0.945 |
